# Identification and functional characterization of an AMD associated c-ABL binding SNP streak within the ARMS2 gene promoter region

**DOI:** 10.1101/2025.10.27.684937

**Published:** 2025-10-27

**Authors:** Ping-Wu Zhang, Sheng Liu, Weifeng Li, Laura Fan, Sean Li, Zi-He Wan, Cynthia A. Berlinicke, Shannath L. Merbs, Donald J. Zack

**Affiliations:** 1. Department of Ophthalmology, Stem Cell Ocular Regenerative Medicine Center, Wilmer Eye Institute, Johns Hopkins University School of Medicine; Baltimore, MD, 21231, USA; 2. Department of Medical and Molecular Genetics, Center for Computational Biology and Bioinformatics, Indiana University School of Medicine, Indianapolis, IN, 46202, USA; 3. Department of Ophthalmology, University of Virginia School of Medicine, Charlottesville, VA, 22908, USA; 4. Solomon H. Snyder Department of Neuroscience, Department of Molecular Biology and Genetics, Department of Genetic Medicine, Center for Nanomedicine at the Wilmer Eye Institute, The Johns Hopkins University School of Medicine, Baltimore, Maryland, United States

**Keywords:** Age-related macular degeneration (AMD), *ARMS2*, single-nucleotide polymorphism (SNP), c-ABL, SNP streak, genetic association, Protein binding SNP, Cis- and trans-acting element

## Abstract

**Background:**

Large-scale genome-wide association studies (GWAS) have identified the human 10q26 locus as a major genetic risk factor for age-related macular degeneration (AMD). The AMD-associated interval has been refined to a 5,196 bp segment flanking the *ARMS2–HTRA1* region, excluding HTRA1 and the ARMS2 3′ indel (443del54ins) variant by risk haplotype analysis. Although the missense SNP rs10490924 has been proposed as a functional variant, its role in AMD remains controversial, and the causative variants and underlying mechanisms within this region remain unresolved.

**Methods:**

An unbiased bioinformatic screen identified a 5-SNP block within the 5,196 bp interval that potentially alters c-ABL protein binding. Protein–DNA interactions were validated using electrophoretic mobility shift assay (EMSA) and chromatin immunoprecipitation (ChIP) assays. Genetic association with AMD (dry and wet subtypes) was assessed in patient cohorts using blood genomic DNA. The regulatory effect of the 5-SNP block was further examined using luciferase reporter assays.

**Findings:**

We identified a 5-SNP block located ~556 bp upstream of the ARMS2 start codon, representing a cluster of predicted c-ABL tyrosine kinase binding sites. This block, in complete linkage disequilibrium with rs10490924 (A69S), showed a strong association with both wet and dry AMD (136 controls, 179 dry AMD, 251 wet AMD). EMSA and ChIP confirmed direct c-ABL binding, while luciferase reporter assays demonstrated reduced transcriptional activity mediated by the 5-SNP block in the presence of c-ABL.

**Interpretation:**

Our results suggest that the c-ABL–responsive 5-SNP regulatory streak in the ARMS2 promoter region act as functional non-coding elements that may contribute to AMD pathogenesis through altered transcriptional regulation.

## Introduction

Age-related macular degeneration (AMD), a leading cause of irreversible blindness in elderly populations, is characterized by progressive degeneration of photoreceptor (PR) and the retinal pigment epithelial (RPE) cells in the macula region. Clinically, AMD manifests as two major forms: wet (neovascular) AMD, which accounts for 10–15% of cases and is more severe, and dry (atrophic) AMD, which represents 85–90% of cases. By 2040, the global prevalence of AMD is projected to rise to nearly 288 million individuals [1]. While anti-VEGF therapies have transformed the management of wet AMD, effective treatments for dry AMD remain limited. Recently developed complement-targeting therapies (C3 and C5 inhibitors) offer promise, but their efficacy remains controversial [2].

AMD arises from a complex interplay of environmental and genetic factors. Aging, smoking, and diet are established non-genetic risk factors, while genetic studies have consistently highlighted two susceptibility loci: 1q31 (CFH) and 10q26 (ARMS2–HTRA1) [3–6]. Despite strong associations across diverse populations, the precise causative variants and pathogenic mechanisms at the 10q26 locus remain unresolved [10,11]. The missense SNP rs10490924 (p.A69S) in ARMS2 and rs11200638 in the HTRA1 promoter have been studied extensively, but strong linkage disequilibrium across the region complicates determination of causality [7–9]. Copy number variation analyses have excluded large structural variants, suggesting that functional contributions most likely arise from SNPs or small indels [12,13].

Functional studies have produced conflicting results. ARMS2, a primate-specific gene, has no clear phenotype in transgenic mice [14,15]. HTRA1-knockout mice also show no retinal changes at 12 months [16], though overexpression of HTRA1 has been linked to pathology in models of polypoidal choroidal vasculopathy and zebrafish photoreceptor degeneration [17,18]. Additional complexity arises from isoform-specific regulation; for example, ARMS2-B is expressed at levels nearly 30-fold higher than ARMS2-A [19]. Studies of ARMS2 and HTRA1 expression in AMD patient tissues have further yielded inconsistent findings [20].

Refinement of the 10q26 risk locus has provided new clues. Fritsche et al. mapped the association to a 30 kb interval upstream of ARMS2, excluding the ARMS2 p.R38X variant [21]. Grassmann et al., analyzing 16,144 AMD cases and 17,832 controls, narrowed the risk haplotype to a 5,196 bp segment encompassing the ARMS2 promoter and first two exons, while excluding the HTRA1 promoter and ARMS2 3′ indel variant [22]. These results suggest the presence of as-yet unidentified regulatory elements in this region.

Taken together, the evidence points toward noncoding regulatory variants as drivers of risk at the 10q26 locus. To address this gap, we combined genomics, bioinformatics, and functional assays to systematically investigate variants within the 5,196 bp ARMS2 promoter region, with a particular focus on SNPs that may modulate protein–DNA interactions. Our goal was to uncover novel regulatory elements that contribute to AMD pathogenesis.

### Ethics statement

All aspects of this study were conducted in accordance with the principles of the Declaration of Helsinki, with informed consent being obtained from all participants. This project was approved by the Johns Hopkins University School of Medicine Institutional Review Board (IRB).

## Material and Methods

### RNA extraction from cultured cells

Total RNA was extracted using the PureLink RNA Mini Kit (Invitrogen, USA) with on-column DNase digestion, following the manufacturer’s protocol. Samples were processed with chloroform extraction and ethanol precipitation. RNA was purified on spin cartridges and eluted in RNase-free water. RNA concentration and purity were assessed with a NanoDrop spectrophotometer (Thermo Fisher Scientific, USA), and only samples with an A260/280 ratio between 1.7 and 2.1 were used for downstream assays.

### Reverse transcription and quantitative -PCR

Complementary DNA (cDNA) was synthesized from total RNA using either the High-Capacity cDNA Reverse Transcription Kit (Applied Biosystems) or the iScript^™^ cDNA Synthesis Kit (Bio-Rad). Quantitative PCR (qPCR) was performed using iTaq Universal SYBR Green Supermix (Bio-Rad) or SsoAdvanced Universal SYBR Green Supermix (Bio-Rad) on a Bio-Rad CFX-384 Real-Time PCR system. Each reaction was performed in biological triplicates. Gene expression was normalized to the geometric mean of two housekeeping genes (GAPDH and ACTB). Primer sequences are provided in Table S2.

### Genomic DNA extraction

Genomic DNA (gDNA) was isolated using the Quick-DNA MicroPrep Kit (Zymo Research, USA). Human eye tissues (5–10 mg, fresh or frozen) were homogenized in 500 μL Genomic Lysis Buffer (Quick-DNA Microprep Kit, Zymo, USA and SCILOGEX D160 homogenizer) and digested with proteinase K (final concentration 1 mg/mL; Invitrogen, USA) at 60 °C for 2–3 h. Lysates were centrifuged at 10,000 × g for 5 min, and the supernatant was applied to Zymo-Spin IC columns. Wash steps were performed sequentially with DNA Pre-Wash Buffer (200 μL) and gDNA Wash Buffer (500 μL), each followed by centrifugation at 10,000 × g for 1 min. DNA was eluted in 50 μL DNA elution buffer after 5 min incubation at room temperature and recovered by centrifugation at 10,000 × g for 1 min.

### Sanger sequencing

DNA sequencing was performed at the Johns Hopkins Genetics Resources Core Facility using an Applied Biosystems 3730xl DNA Analyzer (Thermo Fisher Scientific, USA).

### Bioinformatic prediction of SNP–protein interactions

Within the 5,196 bp region (chr10:122,453,933–122,454,174, GRCh38), 27 common SNPs (minor allele frequency >0.1) were analyzed. For each SNP, 30 bp flanking sequences were scanned using TFBSTools [23] and 6,088 motifs curated from published studies and the MotifDB database [24]. Motif enrichment was filtered using a Benjamini–Hochberg adjusted p-value <1 × 10^−3^. Transcription Factor Affinity Prediction (TRAP) [25–27] was then applied to calculate motif affinities for both reference and alternate alleles. Only motifs with affinity p-values <1 × 10^−4^ and significant allele-dependent changes were retained.

### Electrophoretic mobility shift assay (EMSA)

A PCR fragment of 220 bp covering the 5-SNP streak was used to test the binding between c-ABL protein and the 5-SNP streak using a published method [28]. The 220 bp PCR fragment was amplified using primers: forward 5′-TGAGGCCACAATAAGCTGTG-3′ and reverse 5′-GGTAAGCATTCGGGAAATCTG-3′. PCR cycling conditions were: 98 °C for 1 min, followed by 30 cycles of 98 °C for 10 s, 61 °C for 10 s, and 72 °C for 20 s. Binding reactions contained 1.91 × 10^−9^ M DNA (~100 ng) and increasing concentrations of recombinant human c-ABL protein (0.08–1.32 × 10^−8^ M; ab136358, Abcam, UK) in binding buffer (10 mM Tris, pH 7.6, 50 mM KCl, 1 mM DTT, 10 μg/mL BSA). Reactions were incubated on ice for 30 min and resolved on 1.5% agarose gels at 100 V for 50 min.

### Genotyping of SNP rs10490924 and the 5-SNP block

SNP rs10490924 was genotyped by PCR amplification (191 bp product), followed by restriction digestion with PvuII and agarose gel electrophoresis. Wild-type homozygous G/G alleles produced two fragments (95 bp and 96 bp), homozygous T/T alleles remained uncut (191 bp), and heterozygotes displayed all three bands.

The 5-SNP block was genotyped by Sanger sequencing of a 1.1 kb PCR product amplified with primers: forward 5′-GCACTTTTGCAGGTAGATTGC-3′ and reverse 5′-CAGTGTCAGGTGGTGCTGAG-3′. The wild-type allele sequence was AACAACAACAACAAAAAAACAACAA, and the mutant allele was AAAAAAAAAAAAAAAAAAAAAACAA.

### Cell culture

ARPE-19 cells were maintained at 37 °C in a humidified atmosphere (5% CO_₂_, 95% air) in Dulbecco’s modified Eagle’s medium (DMEM; 4.5 g/L glucose, L-glutamine, pyruvate) supplemented with 1% heat-inactivated fetal calf serum (Sigma Aldrich, USA) and 1% penicillin/streptomycin (Sigma Aldrich, USA).

### Chromatin immunoprecipitation (ChIP) assay

Cells were cross-linked with 1% formaldehyde in DMEM/10% FBS for 30 min at room temperature, quenched with 0.125 M glycine, and washed three times with ice-cold PBS containing 0.5 mM PMSF. Nuclei were isolated, resuspended in sonication buffer, and sonicated (VibraCell Sonicator, USA) 12 times for 10 s each. Lysates were pre-cleared with Protein A Sepharose beads and incubated overnight with 5 μg of antibody, followed by Protein A Sepharose capture. Immunoprecipitates were washed sequentially with buffers A, B, and TE, eluted in 200 μL elution buffer, and DNA was purified by phenol/chloroform extraction and ethanol precipitation with glycogen and sodium acetate. Enriched DNA was analyzed by PCR.

### Luciferase reporter constructs and assay

A 2.3 kb fragment upstream of ARMS2 was amplified from non-AMD genomic DNA (rs10490924 GG genotype) and cloned into the pGL3-basic vector (Promega) using KpnI/XhoI restriction sites. A 300 bp fragment carrying either the wild-type (AACAACAACAACAAAAAAACAACAA) or mutant (AAAAAAAAAAAAAAAAAAAAAACAA) 5-SNP sequence was substituted using Gibson Assembly (New England Biolabs, USA).

HEK293 and ARPE-19 cells were cultured in DMEM/10% FBS for 2 days and 10 days, respectively, before transfection. Plasmid transfections were performed using Lipofectamine^™^ 2000 (Thermo Fisher Scientific, USA). After 48 h, cells were lysed, and luciferase activity was measured using the Luciferase Assay System (Promega, USA). Luminescence was normalized to protein content.

### Statistical analysis

All data were analyzed using GraphPad Prism (GraphPad Software, USA) and Microsoft Excel. Statistical significance was evaluated using Student’s *t*-test or chi-square test, as appropriate. *P* < 0.05 was considered significant.

## Results

### Bioinformatic screening identifies c-ABL as a candidate regulator within the 5,196 bp AMD-associated region

**Figure 1.**
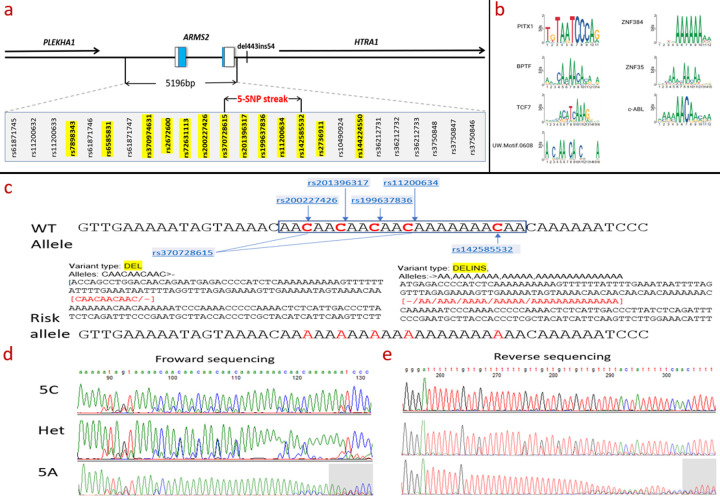
Consensus sequence of top 7 hits and 5 neighboring-SNP Streak sequences. **(A)** DNA sequence highlighting SNPs found in NCBI website are listed. **c**, Rs370728615 and rs142585532 were previously marked as deletion-insertion (DELINS) by NCBI. The figure indicates that all SNPs are C/A SNPs. **(B)** Sequence logos of 7 motifs of binding proteins. Sequence logo is a graphical representation of the DNA binding motif of a transcription factor, based on the information content of each position. The information content ranges from 0, which corresponds to no consensus at the position, to 2, which corresponds to 1 consensus base used at the position. **b, d, e,** Forward and reverse Sanger sequencing reads of homozygous 5C (AA**C**AA**C**AA**C**AA**C**AAAAAAA**C**), homozygous 5A Streaks (AA**A**AA**A**AA**A**AA**A**AAAAAAA**A**) **and** Heterozygous (Het, 5C/5A).

The AMD-associated 5,196 bp interval may contain either previously unrecognized genes/lncRNAs or functional regulatory variants. To investigate the latter possibility, we examined SNPs that could alter protein–DNA interactions within this region. Analysis of the 1000 Genomes Project data identified 229 SNPs spanning rs61871745 (Chr10:124,210,369) to rs3750846 (Chr10:124,215,565), a region in near-complete linkage disequilibrium with the established risk SNP rs10490924 (p.A69S; minor allele frequence (MAF) = 0.273). Because SNPs in strong LD typically share similar allele frequencies [29], we focused on common variants (MAF > 0.1), narrowing the list to 25, plus two flanking SNPs (27 total). Thirteen of these had been previously reported by Grassmann et al., while 12 were newly identified in our analysis (Figure 1A).

Bioinformatic screening, combining motif scanning with binding free energy predictions, identified seven candidate trans-acting proteins, primarily transcription factors and kinases, whose binding may be influenced by SNP variation (Figure 1B). Among them, c-ABL (Abelson non-receptor tyrosine kinase) was the top candidate, supported by both altered binding free energy and recurrent motif enrichment (Table 1). The consensus c-ABL motif (5′-AA/CAACAAA/C-3′ [30]) was densely clustered in a 28 bp segment (Chr10:122,454,671–122,454,688), ~550 bp upstream of the ARMS2 start codon. This region harbors multiple tandem motifs (AACAACAACAACAAAAAAACAACAA) that overlap five consecutive SNPs, suggesting that allelic variation within this “5-SNP streak” could disrupt c-ABL binding and thereby influence transcriptional regulation relevant to AMD.

### Characterization of a functional 5-SNP streak upstream of ARMS2

The c-ABL-binding segment contained six consecutive SNPs, according to NCBI annotations. Four SNPs (rs200227426, rs201396317, rs199637836, rs11200634) shared an identical MAF of 0.273 in the 1000 Genomes Project, while rs370728615 and rs142585532 lacked reliable MAF data. To clarify variant status, we performed Sanger sequencing in 78 human DNA samples (24 controls, 23 wet AMD, 31 dry AMD). This analysis differed from NCBI annotations in that rs370728615 and rs142585532 appear as C/A SNPs rather than as deletion–insertion variants (Figure 1C).

Bidirectional sequencing was necessary to overcome limited read quality in the C-rich downstream flanking region (Figure 1D). These experiments confirmed the presence of five tightly linked SNPs - rs200227426, rs201396317, rs199637836, rs11200634, and rs142585532 - consistently forming a haplotype streak. This 5-SNP streak existed either as a wild-type allele (5C: AACAACAACAACAAAAAAACAA) or as a mutant allele (5A: AAAAAAAAAAAAAAAAAAAAAA). Four of these SNPs had identical allele frequencies in 1000 Genomes, consistent with their strong LD. Together, these results defined a 5-SNP regulatory haplotype in the $ARMS2$ promoter region.

### The 5-SNP streak is strongly associated with AMD

The 5-SNP streak we identified, located approximately 550 bp upstream of the ARMS2 start codon, is not included among the 13 SNPs previously associated with AMD in the 5,196 bp region. Although this upstream region contains SNPs in strong linkage disequilibrium (LD) with rs10490924, this does not imply that all SNPs in the region share high LD with it.

We next evaluated whether the 5-SNP streak is associated with AMD susceptibility. Genotyping in 438 AMD patients (179 dry AMD, 251 wet AMD) and 136 controls revealed that the mutant 5A streak was significantly associated with both dry AMD (OR = 3.37; 95% CI: 2.35–4.84; p = 1.87 × 10^−11^) and wet AMD (OR = 3.80; 95% CI: 2.70–5.35; p = 3.28 × 10^−15^) (Table 2). Further analysis demonstrated that the 5-SNP streak is in near-complete LD with rs10490924 (r^2^ = 0.99; n = 514 samples), reinforcing its candidacy as a functional regulatory element and a surrogate genetic marker for AMD risk.

### Experimental validation confirms direct c-ABL binding to the 5-SNP streak

**Figure 2.**
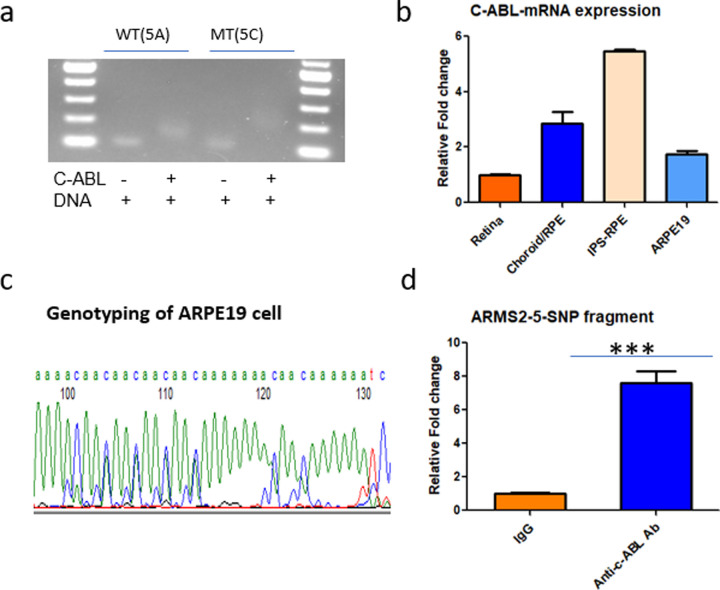
Verification of the binding of C-ABL transcription factor binding with the AMD-associated 5-SNP Streak. **a,** EMSA shows C-ABL varied binding to the ARMS2-5-SNP fragment in human donor samples with different haplotypes: 5A (risk) and 5C (non-risk). **b,** Quantification of ChIP in ARPE19 cells using an anti-C-ABL antibody vs. control IgG shows significant enrichment at the 5-SNP region (***p < 0.001). **c**, Expression levels of C-ABL mRNA in different ocular cell types/tissues by qPCR, showing higher expression in iPSC-derived RPE and choroid/RPE compared to retina and ARPE19 cells. **d**, Representative sequencing trace showing genotyping of the ARPE19 cell line at the 5-SNP locus, confirming its allelic configuration.

To test whether c-ABL binds this region, we performed EMSA using recombinant c-ABL protein. The 220 bp fragment with the mutant 5A allele demonstrated a distinct mobility shift against the wild-type 5C allele, validating allele-specific c-ABL binding. (Figure 2A).

Using ARPE-19 cells (verified as heterozygous at the 5-SNP streak by sequencing; Figure 2C), we performed ChIP. Quantitative PCR (qPCR) following ChIP with anti–c-ABL antibody revealed a 7.6-fold enrichment of the wild-type 5C allele DNA fragments compared with the IgG control, confirming in vivo c-ABL occupancy at the locus (Figure 2D).

To verify c-ABL relevance in ocular contexts, we measured c-ABL mRNA expression. Transcripts were detected in human retina, choroid/RPE, iPSC-derived RPE, and ARPE-19 cells, with higher levels in choroid/RPE and iPSC-derived RPE compared to retina and ARPE-19 (Figure 2B).

### c-ABL binding represses transcriptional activity through the 5-SNP streak

**Figure 3.**
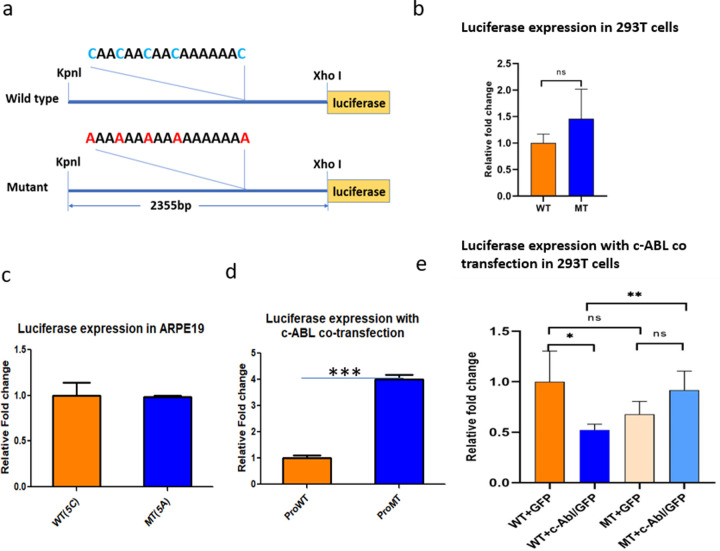
Functional analysis of the 5-SNP streak. (A) Schematic diagram of the wild-type (WT) and mutant (MT) 5-SNP Streak cloned into a luciferase reporter construct. WT allele carries the canonical CA/CA repeat sequence, while the mutant allele contains an A/A tract. Restriction sites (KpnI, XhoI) used for cloning are indicated. (B) Luciferase reporter assay in HEK293T cells showing relative transcriptional activity of WT and MT constructs. No significant difference (ns) was observed. (C) Luciferase assay in ARPE-19 cells transfected with WT or MT constructs, demonstrating similar activity between the two alleles. (D) Co-transfection of HEK293T cells with c-ABL significantly increased luciferase activity from the MT construct compared to WT, indicating allele-specific responsiveness to c-ABL (***P < 0.001). (E) Comparison of luciferase expression in HEK293T cells co-transfected with GFP or c-ABL–GFP together with WT or MT constructs. WT constructs showed a significant increase in reporter activity in the presence of c-ABL (*P < 0.05, **P < 0.01), while MT constructs did not show c-ABL responsiveness. Data represent mean ± SEM from three biological replicates.

To determine functional consequences, we cloned a 2.3 kb ARMS2 upstream fragment containing either the 5C (wild-type) or 5A (mutant) streak into luciferase reporter constructs (Figure 3A). Baseline reporter assays in HEK293T and ARPE-19 cells showed no significant differences between the 5C and 5A alleles (Figure 3B–C), consistent with previous findings [31].

However, allele-specific effects emerged upon co-transfection with a c-ABL expression plasmid. c-ABL overexpression significantly suppressed the wild-type 5C construct’s luciferase activity, while the mutant 5A construct was resistant to this repression (Figure 3D–E). Specifically, the 5A construct’s luciferase activity showed an increase of about 2-fold in HEK293T and 4-fold in ARPE-19 cells compared to the 5C construct. These results indicate that c-ABL binding to the 5C allele functions as a transcriptional repressor, while the 5A allele abrogates repression.

## Discussion

The 10q26 risk haplotype has consistently emerged as one of the strongest genetic signals for AMD, refining the susceptibility interval to the *ARMS2–HTRA1* locus. This has led to speculation that ARMS2 may represent the true causal gene or that the critical regulatory elements reside within its promoter. However, genetic association alone cannot establish causality, and the precise variants driving AMD risk remain unresolved. Most variants within this region, including the well-studied missense SNP rs10490924 (p.A69S), have uncertain functional consequences, and their pathogenic relevance continues to be debated [10].

Previous investigations have primarily focused on cis-regulatory mechanisms and protein-coding functions of *ARMS2* and *HTRA1*. Although cis-effects within the 5,196 bp promoter region have been reported, the possibility of trans-regulatory influences has been largely overlooked. Given the density of associated SNPs—25 common variants within this short interval—it is plausible that multiple variants act in concert to influence gene regulation, keeping both *ARMS2* and *HTRA1* as viable candidates for AMD pathogenesis.

In this study, we identified c-ABL as the strongest candidate trans-acting protein interacting with a 5-SNP block in the ARMS2 promoter region. Originally characterized as the mammalian homolog of the Abelson murine leukemia virus oncogene [32], c-ABL is a non-receptor tyrosine kinase with key roles in DNA damage responses, oxidative stress signaling, and neuronal cell death [33]. Our in vitro analyses demonstrated that this noncoding 5-SNP streak can modulate transcriptional activity in a c-ABL–dependent manner, supporting a model in which sequence variation disrupts repressor binding and thereby alters gene expression.

These findings expand current understanding of the 10q26 locus by implicating a trans-acting regulatory mechanism in addition to previously reported cis-effects. Nonetheless, limitations remain. Our binding screen was restricted to transcription factors and kinases catalogued in existing motif databases, and additional relevant regulators may have been missed. Moreover, while our assays demonstrate binding and functional effects, the downstream gene targets and the tissue-specific consequences of altered c-ABL binding remain to be elucidated.

In summary, we define a novel 5-SNP regulatory streak upstream of ARMS2 that directly binds c-ABL and influences transcriptional regulation. This discovery highlights noncoding elements as potential drivers of AMD risk and provides a framework for future studies aimed at pinpointing causal variants and mechanistic pathways. Ultimately, resolving the functional impact of these variants may uncover new molecular targets and therapeutic strategies for AMD.

## Figures and Tables

**Table 1.** C-ABL is the candidate for a top trans-acting factor whose binding may be affected by SNP polymorphism in the 5k region

	SNP.name	GRCh37 position	GRCh38 position	Alleles	Frequency	Variation type	Binding free energy (p<10^−4^)	Free energy affected by polymorphism (p<10^−4^)	Motif binding match score (adj. p<10^−3^)
Upstream	rs2223089	10:124210160	10:122450644	G/C	0.129 (C)	SNP			
First SNP	rs61871745	10:124210369	10:122450853	G/A	0.286 (A)	SNP			
	rs11200632	10:124211536	10:122452020	A/G	0.286 (G)	SNP			
	rs11200633	10:124211596	10:122452080	C/T	0.286 (T)	SNP			
	rs7898343	10:124212887	10:122453371	G/C	0.135 (C)	SNP			
	rs61871746	10:124212913	10:122453397	T/C	0.287 (C)	SNP			
	rs6585831	10:124212945	10:122453429	T/C	0.356 (T)	SNP			
	rs61871747	10:124213046	10:122453530	C/T	0.287 (T)	SNP			PITX1
	rs370974631	10:124213144	10:122453628	AA/-	0.304 (−)	deletion	ZNF384	ZNF384	BPTF, ZNF35, ZNF384
	rs2672600	10:124213239	10:122453723	G/C	0.349 (G)	SNP			
	rs72631113	10:124213449	10:122453933	C/T	0.402 (T)	SNP			CABL, BPTF, ZNF35
	rs200227426	10:124213671	10:122454155	C/A	0.273 (A)	SNP	CABL	CABL, UW.Motif.0608	CABL, BPTF, TCF7, ZNF35
	rs370728615	10:124213671	10:122454155	CAACAACAAC/-	-	deletion	CABL	CABL	CABL, BPTF, TCF7, ZNF35
	rs201396317	10:124213674	10:122454158	C/A	0.273 (A)	SNP	CABL	CABL	CABL, BPTF, TCF7, ZNF35
	rs199637836	10:124213677	10:122454161	C/A	0.273 (A)	SNP	CABL	CABL	CABL, BPTF, TCF7, ZNF35
	rs11200634	10:124213680	10:122454164	C/A	0.273 (A)	SNP	CABL	CABL	CABL, BPTF, TCF7, ZNF35
	rs142585532	10:124213689	10:122454173	-/AAAAAAAAAAA AAA	-	insertion	CABL, ZNF384	UW.Motif.0608	CABL, BPTF, TCF7, TNF35
	rs2736911	10:124214355	10:122454839	C/T	0.114 (T)	SNP			
	rs10490924	10:124214448	10:122454932	G/T	0.287 (T)	SNP			
	rs61544945	10:124214601	10:122455085	-/GT	0.287 (GT)	insertion			
	rs36212731	10:124214976	10:122455460	G/T	0.287 (T)	SNP			
	rs36212732	10:124215198	10:122455682	A/G	0.287 (G)	SNP			
	rs36212733	10:124215211	10:122455695	T/C	0.287 (C)	SNP			
	rs3750848	10:124215315	10:122455799	T/G	0.287 (G)	SNP			
	rs3750847	10:124215421	10:122455905	C/T	0.287 (T)	SNP			
Last SNP	rs3750846	10:124215565	10:122456049	T/C	0.292 (C)	SNP			
Down stream	rs2736912	10:124215594	10:122456078	C/T	0.115 (T)	SNP			

**Note:** This table summarizes genetic variants within the 5 kb region near the *ARMS2/HTRA1* locus that may influence transcription factor binding. Variants are listed by SNP name, genomic coordinates (GRCh37 and GRCh38), alleles, and allele frequency. Columns indicate the variation type (e.g., SNP, deletion, insertion), and computational predictions for: Binding free energy (ΔG, in kcal/mol ×10^−4^), Whether polymorphism affects binding free energy, Motif binding match score (adjusted p < 1×10^−6^), alongside the predicted transcription factors (TFs) affected. C-ABL appears frequently as the predicted binding factor whose interaction is most affected by sequence variants. Key SNPs highlighted in yellow are part of the previously defined AMD-associated 5-SNP risk haplotype. Variants with potentially altered C-ABL binding are marked, and motifs disrupted include those for PITX1, BPTF, TCF7, ZNF35, and ZNF384, suggesting a functional role of these polymorphisms in modulating gene regulation.

**Table 2:** The 5-SNP Streak is significantly associated with both wet and dry forms of age-related macular degeneration (AMD)

5-SNP Streak	Non-AMD	Wet AMD			Dry AMD		

Genotype	Control (%)	Cases (%)	OR (95% CI) vs reference	P	Cases (%)	OR (95% CI vs reference)	P
5C homozygous	89 (65.44)	7 (31.8)			63 (24.32)		
5C/5A heterozygous	39 (28.68)	8 (34.8)			138 (53.28)		
5A homozygous	8 (5.88)	8 (34.8)			58 (22.39)		
5C	217 (79.78)	22 (47.8)			264 (50.96)		
5A	55 (20.72)	24 (52.2)	3.37 (2.35–4.84)	1.87e-11	254 (49.03)	3.80 (2.70–5.35)	3.28e-15
Total alleles	272 (100)	358 (100)			518 (100)		
Total samples	136	179			259		

**Note:** Genotypic distribution of the 5C and 5A SNP haplotype Streaks in the non-AMD controls, wet AMD, and dry AMD patients. Genotypes include 5C homozygous, 5C/5A heterozygous, and 5A homozygous individuals. The table shows the number and percentage of individuals within each group, along with odds ratios (ORs), 95% confidence intervals (CIs), and p-values relative to the reference group (non-AMD).

## Data Availability

The authors declare that all data generated or analysed during this study are included in this published article.
